# All-proportional solid solution versus two-phase coexistence in the Ti–V alloy by first-principles phase field and SQS methods

**DOI:** 10.1038/s41598-022-13906-7

**Published:** 2022-06-16

**Authors:** Kaoru Ohno, Riichi Kuwahara, Thi Nu Pham, Swastibrata Bhattacharyya, Ryoji Sahara

**Affiliations:** 1 Graduate School of Engineering, Yokohama National University, 79-5 Tokiwadai, Hodogaya-ku, Yokohama, 240-8501 Japan; 2 Research Center for Structural Materials, National Institute for Materials Science (NIMS), 1-2-1 Sengen, Tsukuba, 305-0047 Japan; 3 Biovia Division, Dassault Systèmes K. K., ThinkPark Tower, 2-1-1 Osaki, Shinagawa-ku, Tokyo, 141-6020 Japan; 4 Department of Physics, Birla Institute of Technology and Science Pilani, K. K. Birla Goa Campus, Zuarinagar, Goa, 403726 India

**Keywords:** Materials science, Mathematics and computing, Physics

## Abstract

The microstructures of the Ti–V alloy are studied by purely first-principles calculations without relying on any empirical or experimental parameter. The special quasirandom structure model is employed to treat the all-proportional solid solution $\beta$ phase, while the first-principles phase field method or its variant is employed to treat the coexistence phases. The linearity of the calculated local free energy against the integer Ti$_n$V$_m$ composition in the cluster expansion method manifests a clear evidence of the solid solution behavior. From a detailed energy comparison, our results are consistent with the experimental fact that the Ti–V alloy is an all-proportional solid solution of the $\beta$ phase at high temperatures and exhibits an $\alpha +\beta$ coexistence at low temperatures. Moreover, it is found that mosaic-type microstructures may appear as a metastable phase, as observed by many experiments. The first-principles criterion for the all-proportional solid solution behavior presented in this paper is very general and can be applied to any other binary or multi-component alloys.

## Introduction

As an empirical criterion whether a binary alloy exhibits solubility, Hume–Rothery rule^1,2^ is famous. As per this rule, for the complete solubility, the two atoms should have (1) the same crystal structure, (2) the atomic radius no more different by 15 %, (3) similar valency, and (4) similar electronegativity. However, the criterion is purely empirical and not very accurate^3,4^. For example, among the alloys known as all-proportional solid solutions, the atomic radius is different by 25% and 22%, respectively, in the Ti–Y and Ti–U systems; the electronegativity is largely different by 0.53 and 0.62, respectively, in the V–Mo and Ti–Mo systems. There is a 17% difference in the atomic radius of the Cu–Au system, and a 0.45 difference in the electronegativity of the Fe-Pt system, both of which are well known to behave as all-proportional solid solutions at high temperatures. Therefore, a more reliable and detailed theoretical or computational analysis is required for each alloy.

Here we consider the Ti–V alloy as a typical example of all-proportional solid solution. The Ti–V alloy is one of the low cytotoxic materials, which has a relatively low Young’s modulus^5^. It behaves as all-proportional solid solution in the high temperature *β* phase, while exhibits a two-phase coexistence of the *α* hcp Ti phase and the *β* bcc Ti–V solid solution phase in the lower temperature region^6,7^. It exhibits twin-like microstructures under quench, rolled or high pressure conditions^8–10^, while almost homogeneous, polycrystalline phases are observed at thermal equilibrium^5^. The good deformability of this metastable *β* phase has attracted interest^9,11,12^. This indicates that there is a fine energy balance between the solid solution and coexistence phases.

So far, for the most stable equilibrium phases, extensive thermodynamic studies have been carried out with the CALPHAD method for the Ti–V binary alloy^13^ and the Ti–V-based ternary alloys^14,15^. However, all these studies are based on the experimental or empirical data. It is a challenge to investigate the phase behavior of the Ti–V alloy from first-principles without empirical or experimental parameter. Concerning the first-principles calculations, Uesugi et al.^16^ studied enthalpies of solutions in the Ti–V alloy, and Chinnappan et al.^17^ studied the phase equilibrium by combining cluster expansion and Monte Carlo methods. More recently, Zhou et al.^18^ performed a molecular dynamics study on the tensile stress. Anyway, the first-principles investigation on the solubility of the metallic alloys is hardly advanced.

The main purpose of the present paper is to present a first-principles criterion for the all-proportional solid solution behavior of alloys. We perform a detailed energy comparison and explore the microstructure and the phase behavior of the Ti–V alloy including its metastable phases purely from first-principles without relying on any experimental or empirical parameters. The key strategy here is to employ the special quasirandom structure (SQS) model^19^ to treat the all-proportional solid solution *β* phase and the first-principles phase field (FPPF) method^20–23^ (or its variant) to treat the coexistence phases, and to compare those free energies. In the FPPF method, the cluster expansion method with atomic vacancies and interstitials^24^, potential renormalization theory to eliminate the freedom of off-lattice displacements^20,25^ and a phase-field model are combined with first-principles calculations. So far, we have applied the FPPF method to Ni–Al^20^, Ti64^21^, Ni–Ti^22^ and Ti–Pt^23^ alloys. However, this is the first time study to compare the free energies obtained by the FPPF method and by the SQS model. Here, we also introduce the continuous version of the FPPF method (continuous phase-field (CPF) model) to describe the $\alpha +\beta$ coexistence phase of the Ti–V alloy. Another purpose of this paper is to demonstrate that the linearity of the calculated local free energy against the integer A$_n$B$_m$ composition in the cluster expansion method manifests a clear evidence of the solid solution behavior of the A-B alloy.

Here we note that there are already mature techniques to predict phase diagrams. The cluster expansion method combined with first-principles calculations is a standard approach to predict phase diagrams, and Monte Carlo simulation or cluster variation method (CVM) allows one to calculate the free energy^26^. Moreover, the SQS model combined with first-principles calculations presents a powerful method to treat disordered phases^27^. These are all well established methods. However, the major difference between these preexisting theories and our method is that our method combines the cluster expansion method with the phase field model, not with the Monte Carlo or CVM. Of course, the method using the SQS model is the same as the previous studies^27^. But, the other methods used to compare the resulting free energies are different. The main advantage of the present method is that we can derive not only the free energy but also the associated microstructure simultaneously.

## Methods

**Figure 1:**
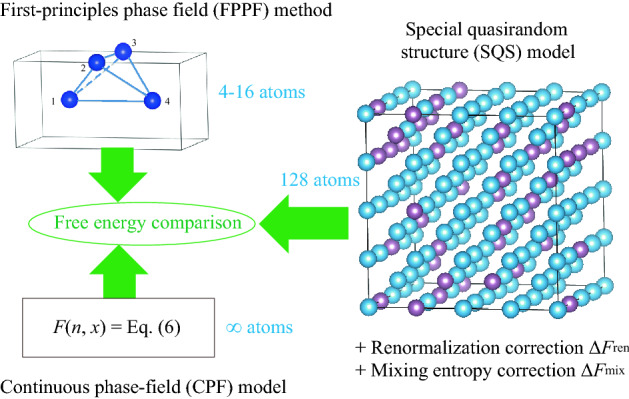
Workflow to compare the free energies evaluated by the FPPF method, the CPF model, and the SQS model.

The FPPF method, the CPF model, and the SQS model are used in this study together with the first-principles method. A detail explanation of each method is given in what follows. The free energies obtained by these methods are compared for various compositions of the Ti–V alloy both at 0 K and 1300 K. The workflow adopting these methods is illustrated in Fig. 1 for easily understanding the present strategy. The purpose of this study is to compare the resulting free energies of three different FPPF, CPF and SQS models. In the FPPF and CPF simulations, we assume mixture of the hcp and bcc phases, while in the SQS simulation, we assume the bcc phase. Different models have different assumptions. Only after the final free energy comparison, we can judge which structure should realize in nature.

### The FPPF method

The space is divided into fine grids, each of which forms a unit cell. We use a tetrahedron approximation in a bcc model (see the upper left panel of Fig. 1) in the cluster expansion method including atomic vacancies^24^. The unit cell is a $\sqrt{2}\times \sqrt{2}\times 1$ supercell of the conventional cubic unit cell and has four lattice points, which can be occupied by either Ti, V, and vacancy. Throughout this paper, we use the bcc lattice constant $a=4.425$ $\mathrm {\mathring{A}}$ obtained by the fully relaxed Ti$_{64}$V$_{64}$ random configuration generated by the SQS model^28,29^. It is well known that the hcp *α* phase is energetically much lower than the beta phase when the V concentration is low. Therefore, for pure Ti, we use the result of our previous analysis for Ti64 alloy^21^. The concentration of the *X* element, $\phi _X$, represents the number of atoms inside the unit cell. That is, we assume that $\phi _\mathrm{Ti}$ ($\phi _\mathrm{V}$) $=0$-1, 1-2, 2-3, 3-4, and 4-5 correspond, respectively, to *n* (*m*) $=0, 1, 2, 3$, and 4 of Ti$_n$V$_m$ (ideal configurations satisfy $n+m=4$, but we consider more general configurations $0 < n+m \le 6$ as well). For the primitive cell calculations based on the tetrahedron approximation, the computational conditions are the same as those in our previous calculation of the Ti64 alloy^21^. (There, we have also compared the local free energy of the (low temperature) *α* (hcp) and (high temperature) *β* (bcc) phases of pure Ti, and confirmed that there is a phase transformation between these phases at certain temperature^21^.) In the FPPF simulation, it is not assumed that the structure is bcc. The alloy is a mixture of the hcp and bcc structures. Only in the SQS model, we assume the bcc structure, although the atomic positions inside the $4\times 4\times 4$ supercell are fully relaxed.

In order to determine the local free energy, we use potential renormalization theory^20,25^. Employing a supercell, we evaluate the total energy $E_j({\varvec{r}}_i)$ where a central atom is displaced around the lattice point ${\varvec{R}}$ and all other atoms are fixed at the surrounding lattice points. Taking account of the symmetry of the lattice, we displace the central atom inside the irreducible wedge of the Wigner–Seitz cell, and calculate the total energy deviation $\Delta E_{ij}=E_j({\varvec{r}}_i)-E_j({\varvec{R}})$ from the perfect crystal without displacement. Here, the suffix *j* denotes the species of the center atom and the suffix *i* denotes the *i*-th grid point around the lattice point ${\varvec{R}}$. Calculating $E_j({\varvec{r}}_i)$ for many ${\varvec{r}}_i$ for each center species $j=$ Ti or V, we evaluate the local partition function to determine the renormalized potential that depends on the temperature, which gives the contribution to the local free energy

1 $$\begin{aligned} \Delta F = - k_\mathrm{B}T\log \sum _j C_j\sum _i\exp \left[ -\frac{\Delta E_{ij}}{k_\mathrm{B}T}\right] , \end{aligned}$$

where $C_j$ is the concentration ratio of the center species $j =$ Ti or V for a given configuration (Ti, V, TiV, TiV$_3$, or Ti$_3$V). Finally, by dividing the result by 4^20^, we obtain the desired renormalized correction ∆*F* in the basic tetrahedron. We add this $\Delta F$ to the total energy $E_j({\varvec{R}})$ of the perfect crystal, in which all atoms were fixed at the lattice points, to obtain the local free energy at 1300 K. In general, explicit phonon calculations as in the Debye model are quite difficult due to the appearance of imaginary frequencies for unstable structures such as a pure BCC Ti or a B2 TiV at equiatomic composition, which may be less stable than a random phase. Therefore, potential renormalization theory has a distinct merit to treat the effect of off-lattice movements of atoms. The vibration entropy is included in the renormalization procedure under the framework of Einstein model, where the atoms can independently vibrate around each lattice point. It is very well known that the specific heat calculated by using the Einstein model is valid at least in high temperature regions including room temperature. The local free energy at 1300 K is calculated by taking the trace of the local partition function and eliminating the degrees of freedom at the length scale smaller than the lattice constant as described above. We use $2\times 2\times 2$ bcc cubic supercell including 16 atoms to obtain the local free energy correction $\Delta F$ for each Ti$_{4-n}$V$_n$ with integer *n*; see also^22^. As well as the total energy, the resulting local free energy becomes a stepwise function of the concentration, i.e., the number of atoms of each element inside one tetrahedron. The renormalization procedure is the same as our previous papers on the FPPF method^20–23^, which can automatically be done by using the Pipeline Pilot protocol^21^.

Then, we write the concentration, the chemical potential, and the mobility of element *X* as $\phi _X$, $\mu _X$, and $M_X$, respectively. The flux is given by ${\varvec{J}}_X = -M_X\nabla \mu _X$, and, from the continuity equation $\partial \phi _X/\partial t = - \nabla \cdot {\varvec{J}}_X$, we derive the Cahn–Hilliard equation

2 $$\begin{aligned} \frac{\partial \phi _X}{\partial t} = M_X\nabla ^2\mu _X + \eta , \end{aligned}$$

where $\eta$ represents the random fluctuation. If one regards the local free energy *F* as a continuous function of the concentration, and assumes that the chemical potential is given by $\mu _X = \partial F/\partial \phi _X$, one obtains the standard Cahn–Hilliard equation^30,31^

3 $$\begin{aligned} \frac{\partial \phi _X}{\partial t} = M_X\nabla ^2 \frac{\partial F}{\partial \phi _X} + \eta . \end{aligned}$$

However, since the local free energy is a step function of the concentration, we have to take difference instead of differentiation with respect to the concentration to obtain the chemical potential. (Note that difference is a more natural definition for the chemical potential.) So, we calculate the chemical potential by^20^

4 $$\begin{aligned} \mu _X = F\left( \phi _X + \frac{1}{2}\right) - F\left( \phi _X - \frac{1}{2}\right) -\varepsilon _X\nabla ^2\phi _X, \end{aligned}$$

where $\varepsilon _X$ represents the gradient energy coefficient. The mobility and the gradient energy coefficient are assumed to be independent on the element. (At least for binary alloys, the interfacial energy is usually the same for the Ti and V sides.) They are absorbed by the time scale and the length scale, respectively, by rescaling the time and space coordinates. Therefore, they are dimensionless. The actual size can be determined by comparing the resulting patterns with experimental microstructures. Our model does not contain interfacial energy anisotropy or elastic energy contribution. However, the FPPF method is different from the standard PF model using the continuous local free energy. It is a distinct characteristic of the FPPF method that the faceted shape appears as a result without introducing any interfacial energy anisotropy or elastic energy contribution.

For the FPPF simulation, we use a 3D model composed of $40\times 40\times 40$ meshes with the periodic boundary condition. The mesh space (*dx*) and the time step (*dt*) are set at 0.27 and 0.000125, respectively. The mobility *M* and the gradient energy coefficient $\varepsilon$, which are regarded as scaling parameters and can be set arbitrary, are set at 1 and 0.01, respectively. The amplitude of the random force is chosen as $\vert \eta \vert =0.5$. We assume initial fluctuations of the composition of $\pm c$ with $c=0.3$ at 22 spot points with radius of 4 meshes. The FPPF simulation code is available on the website^32^, and input files for the FPPF simulation are written in Supplementary Information (SI).

### The CPF model

In this study, the CPF model is specifically designed for the first time to simulate the solid solution phase of a binary alloy. We will see that the local (free) energy has almost a linear dependence on the composition. Therefore, the local (free) energy can be approximately replaced by a linear interpolation between the pure bcc Ti energy *A* and the pure bcc V energy *B* as

5 $$\begin{aligned} F(n) = \frac{(4-n)A + nB}{4}, \end{aligned}$$

where *n* stands for the V composition ($0 \le n \le 4$) of the Ti$_{4-n}$V$_n$ alloy (in a unit cell including one tetrahedron). In order to treat the coexistence of the *α* hcp Ti phase and the *β* bcc random Ti–V phase, we assume that a small fraction *x* ($0 \le x \le 4-n$) is the pure *α* hcp Ti phase. Then, the local (free) energy of the coexistence phase becomes

6 $$\begin{aligned} F(n, x) = \frac{xC + (4-n-x)A + nB}{4} \end{aligned}$$

at a given total Ti composition *n*. Here, *C* is the (free) energy of the *α* hcp Ti. (The local energies of *A*, *B*, and *C* at 0 K are changed to the local free energies by adding the renormalization correction $\Delta F_\mathrm{ren}$ at 1300 K.) To obtain the total (free) energy, we have to add the interfacial energy for the interface between the *α* hcp Ti region and the *β* bcc random Ti–V region, which may be estimated as follows: The volume of the *α* phase compared to the total simulation cell volume is *x*/4, and therefore its surface area must be proportional to $x^{2/3}$. However, the *α* phase does not appear as a single island but would have a lamellar or many-islets structure. In such cases, the number of islets would be proportional to *x*. This is of course an assumption, but anyway the power of *x* must be greater than 1 in the interfacial energy. Otherwise there is no minimum in the total (free) energy. Therefore, the total surface area of the *α* phase would be proportional to $x^{2/3} x = x^{5/3}$. Thus, we have to add $Dx^{5/3}$ to Eq. (6), where *D* is a proportional constant for the interfacial energy. This form of the interfacial energy is not essential at all and can be different.

If we minimize this total (free) energy with respect to *x* at given (fixed) *n*, we have $(5D/3)x_m^{2/3} = (A - C)/4$, and in turn,

7 $$\begin{aligned} x_m = \left[ \frac{3(A - C)}{20D}\right] ^{3/2}. \end{aligned}$$

This is the most suitable fraction of the pure *α* hcp phase in the coexistence. If the interfacial energy coefficient *D* is large, $x_m$ becomes small. On the other hand, if the interfacial energy coefficient *D* is small, $x_m$ becomes large and may be larger than $4-n$. In this case, since *x* cannot exceed $4-n$, *x* must be set equal to $4-n$. That is, the maximum value of *x* is $4-n$ anyway. Here, $x_m = 4 - n$ only gives the boarder for the vanadium concentration *n*, where the pure *α*-Ti fraction *x* is treated as $x = 4 - n$ or $x = x_m$. We assume $D=0.05$ eV corresponding to $x_m=3.38$ at 0 K (3.41 at 1300 K) as a typical value. Introducing the new variable $\phi = n + 0.5$, we define the continuous phase-field (CPF) model, which obeys the Cahn–Hilliard equation

8 $$\begin{aligned} \frac{\partial \phi }{\partial t} = M\nabla ^2\mu , \;\;\;\;\; \mu =F(\phi +0.5)-F(\phi -0.5)-\varepsilon \nabla ^2\phi + \eta , \end{aligned}$$

where *F*(*n*) is given by Eq. (6) with $x=4-n$ for $n>4-x_m$ or with $x=x_m$ for $n<4-x_m$. Note that *F*(*n*) does not explicitly include the interfacial energy term $Dx^{5/3}$ discussed above. The interfacial energy appears as the gradient energy proportional to $\varepsilon$ in Eq. (8). Here, all the detailed settings of the CPF simulation (therefore the input files) are the same as for the FPPF simulation discussed above.

For the CPF simulation also, we use a 3D model composed of $40\times 40\times 40$ meshes as well. All detailed settings of the simulation conditions are the same as those for the FPPF  method mentioned above. The CPF simulation code is available on the website^32^, and its usage is written in Supplementary Information (SI).

### First-principles method and the SQS model

For the first principles calculations, we use the plane-wave pseudopotential code CASTEP^33^. Using the Perdew-Burke-Ernzerhof (PBE) functional in the generalized gradient approximation (GGA) of DFT, we set the cut-off energy for plane waves at 500 eV in the on-the-fly generated ultrasoft pseudopotential. In order to simulate purely random configurations for various compositions, we employ the SQS model^19,28,29^. For this purpose, we assume $4\times 4\times 4$ supercell of the conventional cubic unit cell, which includes 128 atoms. Then, the atomic geometries are fully relaxed with the fixed supercell frame using CASTEP. We have generated several samples and compared their total energies, but the total energy dependence on the sample was of the order of 10 meV/atom. Since the calculation of the renormalization at 1300 K is quite difficult for the random configurations in the $4\times 4\times 4$ supercell, we approximately use the renormalization correction $\Delta F_\mathrm{ren}$ interpolated according to the concentration. We also add the mixing entropy term $\Delta F_\mathrm{mix} = -TS_\mathrm{mix}=k_\mathrm{B}T\{(4-n)\log [(4-n)/4]+n\log (n/4)\}$ per four atoms. Then we can estimate the free energy at 1300 K.

## Results and discussion

### The FPPF method

**Figure 2:**
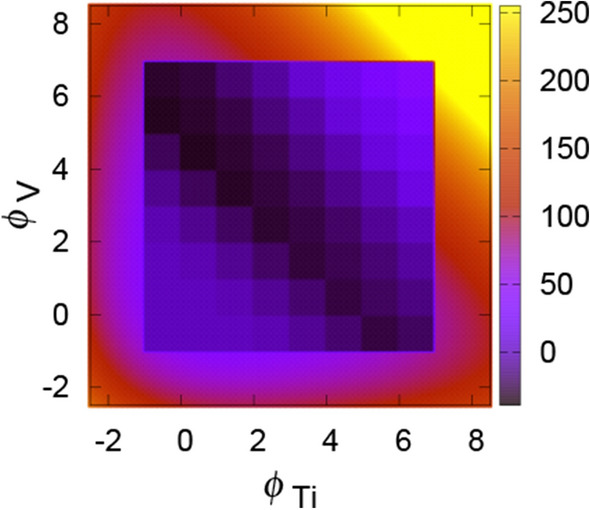
2D map of the local free energy for the original FPPF method. The color scale is in units of eV.

The calculated energy values for the 5 integer compositions of Ti$_n$V$_m$ with $n+m=4$ in the tetrahedron approximation are listed in Table 1 together with the values for the *α* hcp phase^21^. The values inside the parentheses mean the local free energy including the effect of the potential renormalization at 1300 K. At most two interstitial atoms are considered inside the unit cell as well as the atomic vacancies. Its 2D map is shown in Fig. 2. The values for all 27 integer compositions of Ti$_n$V$_m$ with $0 < n+m \le 6$ are given in Table S1 of SI.

**Table 1:** Local energy per four-atom unit cell of each composition in units of eV.

Composition	Structure	Energy (eV)
Ti_4_	hcp	− 27.49 (− 27.41)^a^
Ti_4_	bcc	− 26.70 (− 26.61)
	(supercell)	− 26.74 (− 26.66)
Ti_3_V	bcc	− 28.67 (− 28.59)
Ti_2_V_2_	bcc	− 30.51 (− 30.45)
TiV_3_	bcc	− 32.34 (− 32.27)
V_4_	bcc	− 34.13 (− 34.04)
	(supercell)	− 34.16 (− 34.07)

The values inside the parentheses are the local free energy including the effect of the potential renormalization $\Delta F_\mathrm{ren}$ at 1300 K. The $4\times 4\times 4$ supercell results are also listed for bcc Ti$_4$ and V$_4$.

^a^See Ref. ^21^.

**Figure 3:**
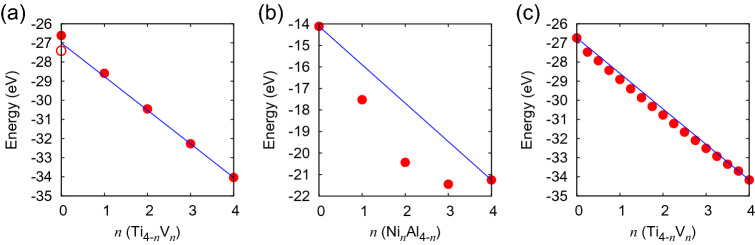
Plots of (**a**) the local free energy versus *n* of Ti$_{4-n}$V$_n$ (the open circle is the energy of hcp Ti, while the solid points are the energies calculated with bcc structure), (**b**) that of Ni$_n$Al$_{4-n}$, and (**c**) the SQS-based energy versus *n* of Ti$_{4-n}$V$_n$. Linear interpolation line is shown in blue color.

The most significant characteristic of the TiV alloy is the linear dependence of the local (free) energy on the V concentration, if we look at the integer Ti$_n$V$_m$ ($n+m=4$) compositions in the tetrahedron 4-atom unit cell; see Fig 3a. This behavior has not been observed in any alloys in our previous studies; NiAl alloy^20^ (see Fig. 3b), TiAl alloy^21^, NiTi alloy^22^, and TiPt alloy^23^. This linear dependence is a distinct characteristic of the low multiple-phase-transformation barrier height or the seamless continuous phase transformations, and is a strong sign of the all-proportional solid solution. In the all-proportional solid solution, the two atoms can be mixed at any composition, and there is no stoichiometric composition. That is, there is no special meaning of the integer compositions like Ti$_4$, Ti$_3$V, Ti$_2$V$_2$, TiV$_3$, and V$_4$ in the tetrahedron model. They are only some passing points in the all-proportional solid solution. This means that at any composition, the resulting local (free) energy should roughly coincide with the arithmetic weighted average between the two extremum compositions, Ti$_4$ and V$_4$, which is indicated by a blue straight line in Fig. 3a. The real plots in between the two extremum compositions are slightly lower than the straight line, reflecting the additional energy gain by the mixing of the two atoms.  So, roughly speaking, we can use this linear dependence of the local (free) energy of the complete A$_n$B$_m$ ($n+m=4$) compositions as a criterion to select the candidates for the all-proportional solid solution. This criterion is rather qualitative and not very deterministic, but we can use  it for the screening purpose.

**Figure 4:**
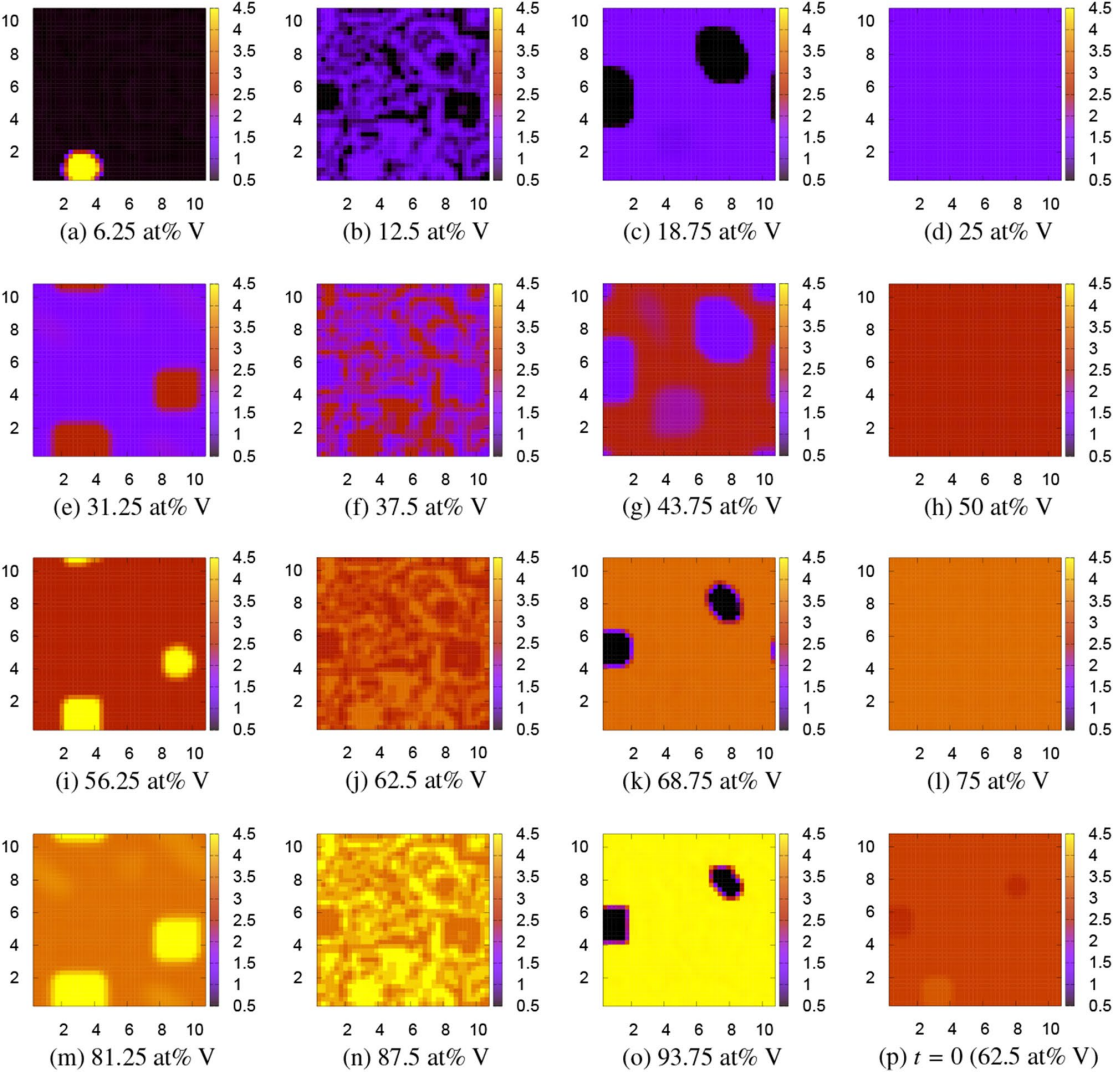
Microstructures of the Ti$_{4-n}$V$_n$ alloy obtained by the first FPPF simulation, FPPF1, using the original tetrahedron approximation at 0 K. The color bar indicates the V concentration $\phi _\mathrm{V}=n+0.5$ ($0.5\le \phi _\mathrm{V}\le 4.5$). The scales in *x* and *y* axes can roughly be regarded as the order of μm. The patterns do not change at all at 1300 K. (p) is the initial pattern at $t=0$ for 62.5 at% V.

Three FPPF simulations are performed for $10^5$ time steps where the microstructure do not change anymore. FPPF1 is the simulation based on the original tetrahedron model with four atoms in the unit cell. The local free energy values, which we used in the tetrahedron model for the FPPF1 simulation, are those of hcp Ti$_4$, bcc Ti$_3$V, bcc Ti$_2$V$_2$, bcc TiV$_3$, and bcc V$_4$. FPPF2 is the simulation with eight atoms in the unit cell, and FPPF3 is the simulation with sixteen atoms in the unit cell. The result of FPPF1 is depicted by Fig. 4. In this figure, the microstructures of the V concentration on the $z=1$ cross section obtained by the FPPF simulation are shown for 15 compositions from (a) 6.25 at% V to (o) 93.75 at% V. Figure 4p shows the initial V concentration in the case of 62.5 at% V. There are striking V precipitates in Fig. 4a of 6.25 at% V or Fig. 4i of 56.25 at% V and Ti precipitates in Fig. 4o of 93.75 at% V or Fig. 4k of 68.75 at% V, which are obviously an artifact of the tetrahedron approximation in a treatment of the Ti–V alloy.

**Figure 5:**
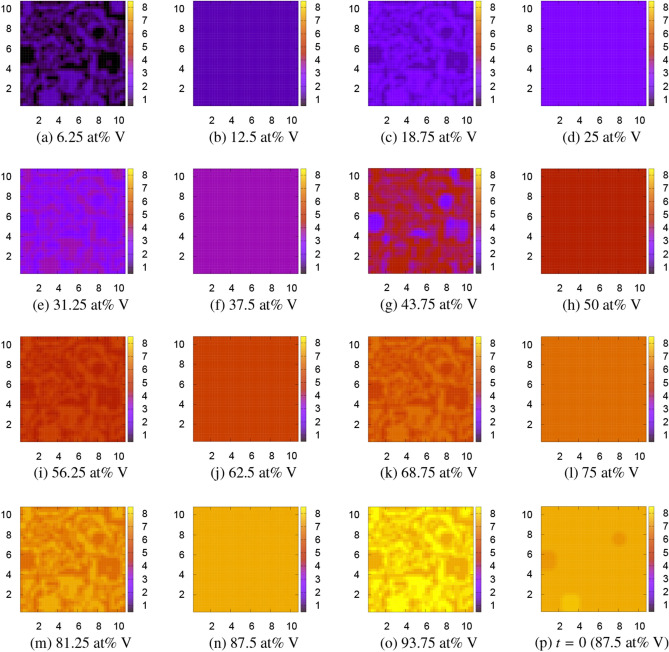
Microstructures of the Ti$_{8-n}$V$_n$ alloy obtained by the second FPPF simulation, FPPF2, using the doubled 8-atom unit cell at 1300 K. The color bar indicates the V concentration $\phi _\mathrm{V}=n+0.5$ ($0.5\le \phi _\mathrm{V}\le 8.5$). The scales in *x* and *y* axes can roughly be regarded as the order of μm. The patterns do not change at all at 0 K. (p) is the initial pattern at $t=0$ for 87.5 at% V.

**Figure 6:**
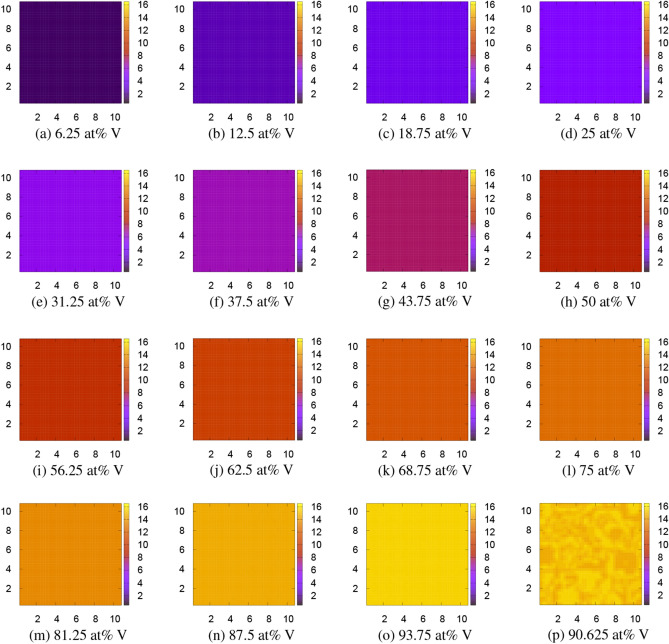
Microstructures of the Ti$_{16-n}$V$_n$ alloy obtained by the third FPPF simulation, FPPF3, using the quadruple 16-atom unit cell at 0 K. The color bar indicates the V concentration $\phi _\mathrm{V}=n+0.5$ ($0.5\le \phi _\mathrm{V}\le 16.5$). The scales in *x* and *y* axes can roughly be regarded as the order of μm. The patterns do not change at all at 1300 K. (p) is the result for the half composition, 90.625 at% V.

Therefore, we doubled the unit cell size to include 8 atoms and performed FPPF2. For the local free energy, we made a simple interpolation between the adjacent concentrations. The result is depicted by Fig. 5. Figure 5p is the initial V concentration in the case of 87.5 at% V. Now, all strange precipitates disappear, and mosaic and homogeneous patterns alternately appear at $6.25+12.5n$ and 12.5*n* at% V for $n = 0, 1, 2, \ldots , 7$. We further doubled the unit cell to include 16 atoms and performed FPPF3, whose result is depicted by Fig. 6. In this simulation, the patterns are homogeneous for all 6.25*n* at% V for $n = 0, 1, 2, \ldots , 16$. However, even in this case, a mosaic pattern appears at 90.625 at% V as shown in Fig. 6p. Although only one example is shown in this figure, all the half compositions of $3.125+6.25n$ at% V have a similar mosaic pattern. These mosaic-type microstructures have a possibility to appear at least in metastable phases. Indeed they very much resemble the experimentally observed twin-like microstructures of metastable phases^8–10^ (compare, for example, Fig. 5 of Ref. ^10^ for 10 at% V with Fig. 4b for 12.5 at% V), although we cannot distinguish these phases with the metastable $\omega$ phase^34–36^ within the present simple analysis. In the FPPF method, the final free energy is evaluated as the spatial integration of the local free energy distribution plus the interfacial energy. The calculated FPPF free energies including the interfacial energy $F_\mathrm{int}=(\varepsilon /4)\sum _{X=\mathrm{Ti, V}}\int [\nabla \phi _X({\varvec{r}})]^2d{\varvec{r}}$ (additional factor 1/2 was multiplied to avoid double counting of Ti and V) at 0 K and at 1300 K are listed in Table 2. The values of the interfacial energy $F_\mathrm{int}$ per four atoms are the order of $1\times 10^{-4}$ eV for homogeneous patterns, about 0.08 eV for mosaic patterns, and 0.004–0.07 eV for patterns with faceted precipitates. The (free) energy obtained by FPPF3 are also written in the seventh and ninth columns of Table 3 for later comparison.

**Table 2:** Total free energy per four atoms obtained by the FPPF1, FPPF2, and FPPF3 simulations at each composition in units of eV.

Concentration	FPPF1	FPPF2	FPPF3	FPPF1	FPPF2	FPPF3
at% V	0 K (eV)			1300 K (eV)		
0.00	− 27.49			− 27.41		
6.25	− 27.80	− 27.70	− 27.78	− 27.71	− 27.62	− 27.71
12.50	− 28.00	− 28.08	− 28.08	− 27.92	− 28.00	− 28.00
18.75	− 28.45	− 28.29	− 28.37	− 28.37	− 28.21	− 28.30
25.00	− 28.67	− 28.67	− 28.67	− 28.59	− 28.59	− 28.59
31.25	− 28.99	− 29.05	− 29.13	− 28.91	− 28.97	− 29.06
37.50	− 29.51	− 29.59	− 29.59	− 29.44	− 29.52	− 29.52
43.75	− 30.21	− 29.97	− 30.05	− 30.14	− 29.90	− 29.99
50.00	− 30.51	− 30.51	− 30.51	− 30.45	− 30.45	− 30.45
56.25	− 30.88	− 30.89	− 30.97	− 30.82	− 30.82	− 30.91
62.50	− 31.35	− 31.43	− 31.43	− 31.28	− 31.36	− 31.36
68.75	− 31.98	− 31.80	− 31.88	− 31.91	− 31.74	− 31.81
75.00	− 32.34	− 32.34	− 32.34	− 32.27	− 32.27	− 32.27
81.25	− 32.65	− 32.70	− 32.79	− 32.58	− 32.63	− 32.71
87.50	− 33.15	− 33.23	− 33.23	− 33.08	− 33.15	− 33.15
93.75	− 33.73	− 33.60	− 33.68	− 33.64	− 33.52	− 33.59
100.00	− 34.16			− 34.07		

The energy values at 0 K are listed in the second to  fourth columns,  while the free energy values at 1300 K are listed in the last  three columns.

### The SQS model

For a reliable judgement whether the target alloy is really the all-proportional solid solution, one should do more precise energy comparison. For this purpose, we performed $4\times 4\times 4$ supercell calculations of the purely random configurations generated by using the SQS model^28,29^ for variety of compositions. Then, the atomic geometries were relaxed with the fixed supercell frame. The resulting energy at 0 K for each composition is listed in the second column (0 K) of Table 3. This energy again has almost a linear dependence on the composition as seen in Fig. 3c. To obtain the free energy at 1300 K, we have to add the renormalization correction $\Delta F_\mathrm{ren}$ and the mixing entropy term $\Delta F_\mathrm{mix}$. Then we obtain the free energy at 1300 K. All these values are listed in the third to fifth columns of Table 3.

**Table 3:** Total free energy per four atoms of each composition in units of eV.

Concentration	Random configurations (eV)				CPF	FPPF3	CPF	FPPF3
at% V	0 K	$\Delta F_\mathrm{ren}$	$\Delta F_\mathrm{mix}$	1300 K	0 K		1300 K	
0.00	− 26.74	0.08	0.00	− 26.66	− 27.49		− 27.41	
6.25	− 27.48	0.08	− 0.05	− 27.44	− 27.84	− 27.78	− 27.77	− 27.71
12.50	− 27.93	0.08	− 0.07	− 27.92	− 28.30	− 28.08	− 28.23	− 28.00
18.75	− 28.44	0.08	− 0.09	− 28.45	− 28.74	− 28.37	− 28.66	− 28.30
25.00	− 28.91	0.08	− 0.11	− 28.95	− 29.16	− 28.67	− 29.08	− 28.59
31.25	− 29.39	0.07	− 0.12	− 29.45	− 29.58	− 29.13	− 29.50	− 29.06
37.50	− 29.87	0.07	− 0.13	− 29.93	− 30.00	− 29.59	− 29.91	− 29.52
43.75	− 30.32	0.06	− 0.13	− 30.39	− 30.41	− 30.05	− 30.33	− 29.99
50.00	− 30.77	0.06	− 0.13	− 30.84	− 30.83	− 30.51	− 30.74	− 30.45
56.25	− 31.22	0.06	− 0.13	− 31.29	− 31.24	− 30.97	− 31.16	− 30.91
62.50	− 31.66	0.06	− 0.13	− 31.73	− 31.66	− 31.43	− 31.58	− 31.36
68.75	− 32.10	0.07	− 0.12	− 32.15	− 32.08	− 31.88	− 31.99	− 31.81
75.00	− 32.51	0.07	− 0.11	− 32.56	− 32.50	− 32.34	− 32.41	− 32.27
81.25	− 32.93	0.07	− 0.09	− 32.95	− 32.91	− 32.79	− 32.83	− 32.71
87.50	− 33.34	0.08	− 0.07	− 33.34	− 33.33	− 33.23	− 33.24	− 33.15
93.75	− 33.74	0.08	− 0.05	− 33.71	− 33.75	− 33.68	− 33.66	− 33.59
100.00	− 34.16	0.08	0.00	− 34.07	− 34.16		− 34.07	

For the purely random configurations, the energy at 0 K, the potential renormalization correction $\Delta F_\mathrm{ren}$, the mixing entropy $\Delta F_\mathrm{mix}=-TS_\mathrm{mix}$, and the free energy at 1300 K are listed in the second to fifth columns. For the results of the CPF and FPPF3 simulations, the energy at 0 K and the free energy at 1300 K are listed in the last four columns.

### The CPF model

**Figure 7:**
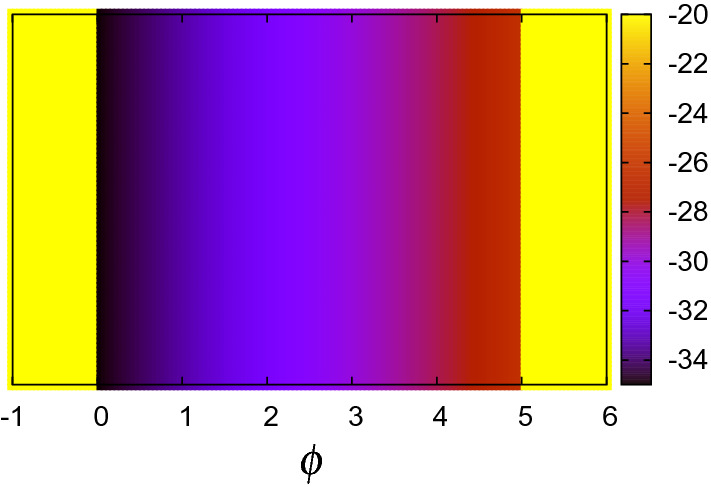
Continuous local energy used for the CPF model at 0 K. Continuous local free energy at 1300 K looks the same, because the potential renormalization correction $\Delta F_\mathrm{ren}$ is very small.

**Figure 8:**
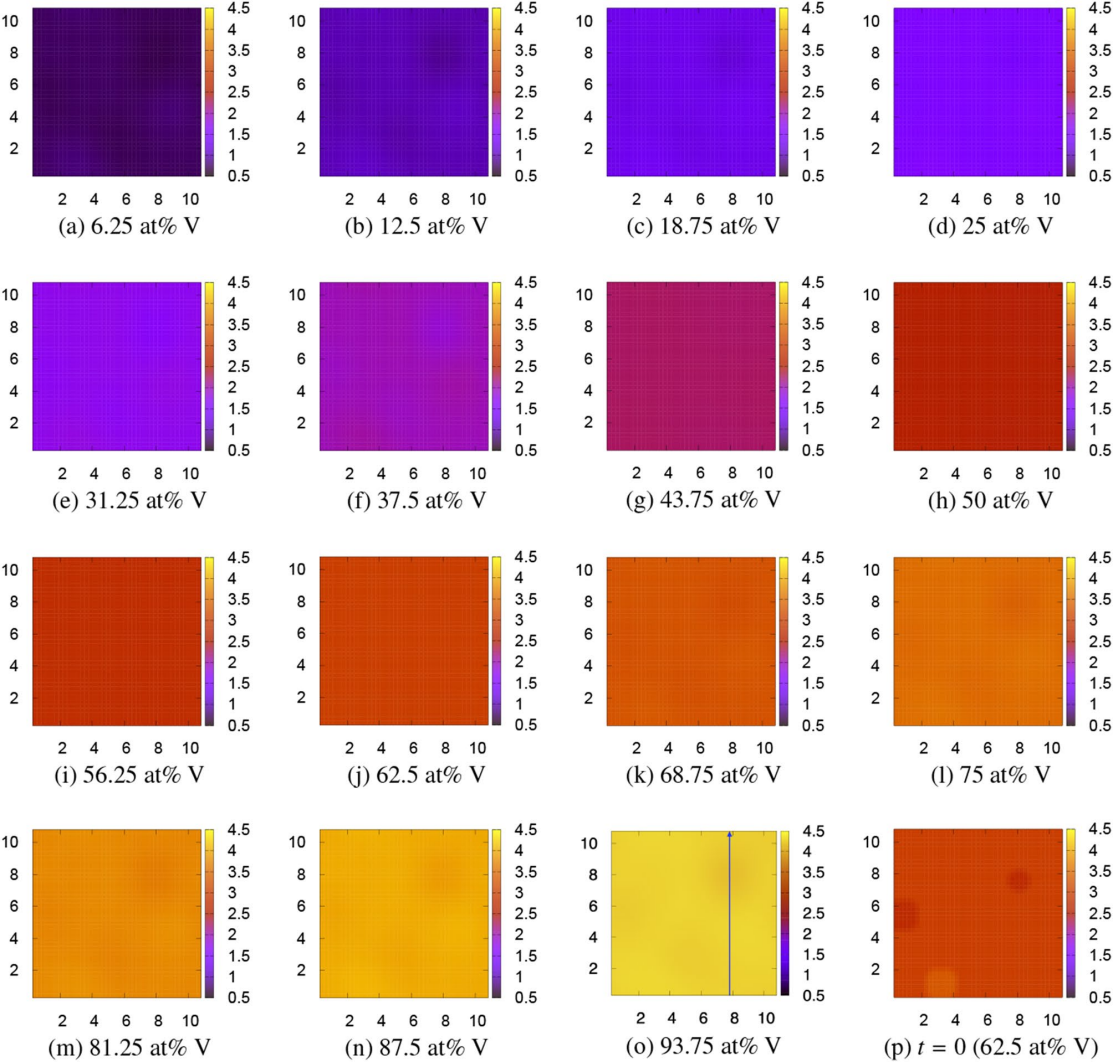
Microstructures of the Ti$_{4-n}$V$_n$ alloy obtained by the CPF model at 0 K. The color bar indicates the V concentration $\phi _\mathrm{V}=n+0.5$. The initial pattern at $t=0$ for 62.5 at% V is shown in (**p**). The scales in *x* and *y* axes can roughly be regarded as the order of μm. The patterns do not change at all at 1300 K. 1D profile along the blue allow of (**o**) 93.75 at% V is depicted in Fig. 9.

**Figure 9:**
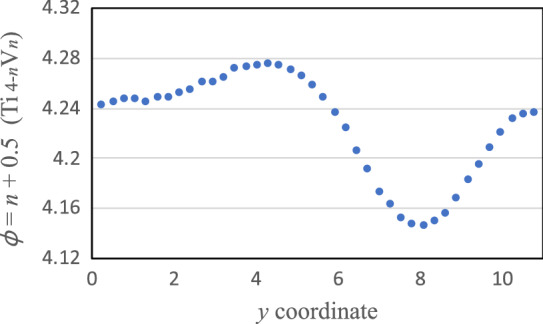
1D profile along the blue allow of Fig. 8o 93.75 at% V.

The local (free) energy of the CPF model, Eq. (6), is shown in Fig. 7. The CPF simulations are performed for $10^5$ time steps where the microstructure do not change anymore. The resulting patterns at 0 K on the $z=1$ cross section are shown in Fig. 8. The initial V concentration is shown in Fig. 8p, which is the same as Fig. 4p. Although there is no clear microstructure, light shading patterns appear for all compositions. 1D profile along the blue allow of (o) 93.75 at% V is depicted in Fig. 9. The V-depletion region around $y=8$ corresponds to the *α* phase and the other region corresponds to the *β* phase. Such a fluctuation in spacial V distributions can be considered as a characteristic of the $\alpha +\beta$ coexisting phase. Since we did not introduce any orientational order parameter^37^ in our model for simplicity and clarity, there is no polycrystal-like domain structure as seen in the experimental patterns^5^. Besides this slight difference, the resulting almost homogenous pattern well resembles the experimental patterns; see, for example, Fig. 1(d)–(e) of Ref. ^5^. There is no evidence of the $\omega$ phase^34–36^ since it appears in metastable states only. There is also no twin-like structure as seen in the experimental patterns under quench, rolled or high pressure^8–10^. In the result of this CPF simulation, the interfacial energy calculated by $(\varepsilon /2)\int [\nabla \phi ({\varvec{r}})]^2d{\varvec{r}}$ is quite small (at most $1\times 10^{-5}$ eV per four atoms) and negligible. The resulting total energy at 0 K and total free energy at 1300 K are summarized in the sixth and eighth columns of Table 3.

### Energy comparison

Now we compare the resulting free energies. From Table 3, we immediately find that the FPPF free energy is higher than the CPF free energy and the free energy of the random configurations for all V compositions at both 0 K and 1300 K, although the difference is not very large but only 0.02–0.59 eV per four atoms. Therefore, the mosaic-type microstructures shown in Fig. 6p may be realized at least as the metastable phases as observed in many experiments^8–10^. Next we compare the CPF free energy with the free energy of the random configurations. At 0 K, the former is lower than the latter for most V concentrations, although the former is the same as or slightly higher than the latter for 62.5–87.5 at% V by 0.01–0.02 eV per four atoms. However, the CPF patterns (Fig. 8) are almost homogeneous and cannot be distinguished from the purely random configurations. Therefore, we conclude that the almost homogeneous $\alpha +\beta$ coexistence phase appears at 0 K. On the other hand, at 1300 K, the CPF free energy is higher than the free energy of the random configurations for most V concentrations, although the former is lower than the latter for 6.25–31.25 at% V by 0.05–0.33 eV per four atoms. It is true that there is an error in the free energy of the random configurations due to the approximation introduced in the renormalization calculation. However, again, the CPF patterns are almost homogeneous and cannot be distinguished from the purely random configurations. Therefore, we can conclude that the almost homogeneous *β* random phase appears at 1300 K. Overall, our results are consistent with the experimental fact that the Ti–V alloy forms the $\alpha +\beta$ two-phase coexistence at low temperatures, and the all-proportional solid solution at high temperatures.

## Concluding remarks

In this paper, we have studied the phase behavior of the Ti–V alloy from purely first-principles without relying on any empirical or experimental parameter. We used the first-principles phase field (FPPF) method and its continuous variant (CPF model) to treat the coexisting phases, and the SQS model to treat the all-proportional solid solution *β* phase. Within the accuracy of the approximation used in the present analysis, the resulting energy comparison successfully derives the experimental fact at phase equilibrium that the Ti–V alloy is an all-proportional solid solution of the *β* phase at high temperatures and exhibits an $\alpha +\beta$ coexistence at low temperatures. Moreover, our result predicts that the mosaic-type microstructures shown in Fig. 6p may be realized at least as the metastable phases as observed by many experiments^8–10^.

We also found that the linear energy dependence of the local free energy on the integer A$_n$B$_m$ ($n+m=4$) compositions in the cluster expansion method can be used as a criterion whether the alloy exhibit a solid solution behavior. Although this criterion is rather qualitative and not very deterministic, it can be used for the screening purpose. We summarize the present general strategy to identify the all-proportional solid solution as follows. First, perform the first-principles total energy calculation of the primitive cell in the first-principles phase field (FPPF) method. Second, perform a very simple screening by checking the linearity of the energy calculated in this method. If this screening is passed, perform first-principles supercell calculations for purely random configurations of the candidate alloy using the SQS model. Then, construct the CPF model if necessary and perform the FPPF and/or CPF simulation. Finally, comparing the resulting energies (or free energies for finite temperatures), identify the most stable microstructure to be either a two-phase coexistence obtained by the FPPF method or a purely random configuration everywhere.

To demonstrate the validity of this  strategy, let us briefly examine the case of the NiAl alloy^20^, which exhibits clear two-phase ($\gamma /\gamma '$) coexistence. First of all, the local free energy of the stoichiometric compositions Ni$_n$Al$_m$ ($n+m=4$) (see Fig. 2 of Ref. ^20^) does not change linearly in *n*; the deviation from the linear interpolation is quite large (2.8 eV at Ni$_2$Al$_2$); see Fig. 3c. Therefore, from the first screening, this alloy is clearly excluded. Furthermore, our $3\times 3\times 3$ cubic fcc supercell calculation of the SQS-based purely random configurations gives the total energies of $-23.54$ eV and $-26.58$ eV, respectively for 50 and 83.3 at% Ni, and the corresponding values for the completely ordered B2 phase, and the L1$_2$ ($-26.26$ eV) and pure Ni fcc ($-27.72$ eV) coexistence phase are $-24.14$ eV and $-26.70$ eV (we added the coexistence interfacial energy of 0.05 eV estimated by the original FPPF  method to the arithmetic weighted average of the L1$_2$ and pure Ni fcc phases), respectively, which indicates that the B2 phase at 50 at% Ni and the two-phase ($\gamma /\gamma '$) coexistence at 83.3 at% Ni are both energetically stable at 0 K. This situation does not change even if we add the mixing entropy of $-\,0.13$ eV and $-\,0.09$ eV, respectively, to the random configurations at 1300 K. Then, assuming that the effect of the potential renormalization at 1300 K is the same both for the uniform random phase and for the coexistence phase, we are led to the conclusion that the coexistence phase is more stable at 1300 K also. This strategy is very general and applicable not only to binary alloys but also to ternary and multi-element alloys. For the FPPF method, we have already succeeded in applying it to ternary alloys like Ti64^21^. To extend the CPF model to ternary alloys is left for future study, but the strategy is straightforward although it depends on case by case. Application of SQS model to ternary alloys is also possible. So, although the application of the present method to ternary alloys is left for future study, there is no essential difficulty in doing it. We believe that the present method is useful for determining the phase morphology of alloys.

## Supplementary Information

Supplementary Information.

## Data Availability

All data generated and analysed during this study are included in the published article and its Supplementary Information.

## Code availability

Code that supports the findings of this study is available on the website^32^.
