# Supplementary material for: All-proportional solid solution versus two-phase coexistence in the Ti–V alloy by first-principles phase field and SQS methods
Source: Sci Rep. 2022 Jun 16;12:10070. doi: 10.1038/s41598-022-13906-7 (PMC9203554; doi:10.1038/s41598-022-13906-7)
Supplement: Supplementary file 1 — Supplementary Information. [file 41598_2022_13906_MOESM1_ESM.pdf]

Supplementary Information

All-proportional solid solution versus two-phase coexistence in the Ti-V alloy by first-principles phase field and SQS methods

Kaoru Ohno[1,2*], Riichi Kuwahara[3], Thi Nu Pham[1], Swastibrata Bhattacharyya[4] and Ryoji Sahara[2]

[1*]Graduate School of Engineering, Yokohama National University, 79-5 Tokiwadai, Hodogaya-ku, Yokohama, 240-8501, Japan.
[2]Research Center for Structural Materials, National Institute for Materials Science (NIMS), 1-2-1 Sengen, Tsukuba, 305-0047, Japan.
[3]Biovia Division, Dassault Systèmes K. K., ThinkPark Tower, 2-1-1 Osaki, Shinagawa-ku, Tokyo, 141-6020, Japan.
[4]Department of Physics, Birla Institute of Technology and Science Pilani, K. K. Birla Goa Campus, Zuarinagar, Goa, 403726, India.

In this Supplementary Information (SI), we present the results for the primitive cell calculations of the TiV alloy based on the tetrahedron approximation. The computational conditions are the same as those in Ref. [1] and all the calculations are automatically done by using the Pipeline Pilot protocol [1]. The calculated local free energy values for the 27 compositions of Ti_nV_m with $1 \leq n+m \leq 6$ in the tetrahedron approximation are listed in Table 1. At most two interstitial atoms are considered inside the unit cell as well as the atomic vacancies. Its 2D map is shown in Fig. 1 of the main paper.

Table 1 Local energy per four-atom unit cell of each composition in units of eV. The values inside the parenthesis are the local free energy including the renormalization at 1300 K

Composition	Energy (eV)	Composition	Energy (eV)
Ti_6	−14.698	Ti_4	−26.695 (−26.612)
Ti_5V	−17.388	Ti_3V	−28.666 (−28.590)
Ti_4V_2	−20.097	Ti_2V_2	−30.510 (−30.451)
Ti_3V_3	−22.086	TiV_3	−32.340 (−32.273)
Ti_2V_4	−24.189	V_4	−34.125 (−34.036)
TiV_5	−26.392	Ti_3	−18.369
V_6	−28.364	Ti_2V	−20.278
Ti_5	−21.669	TiV_2	−21.393
Ti_4V	−24.233	V_3	−22.455
Ti_3V_2	−26.633	Ti_2	−10.227
Ti_2V_3	−28.090	TiV	−10.854
TiV_4	−30.346	V_2	−11.348
V_5	−31.521	Ti	−2.956
		V	−3.214

The simulation code for the FPPF method can be downloaded from the website [2]. It consists of four Fortran 90 programs, FPPFModel.f90, Util_mod.f90, Manager.f90, and Variables.f90. The code is also available in supplementary info. of Ref. [3], but in the subroutine for the 3D Laplacian, NABLA3(A,AL2), of Util_mod.f90 there, a necessary multiplication of dx2_in is missing in the definition of AL2(i,j,k). The code on the website is correct. The code can be compiled by using Makefile. The usage of this code is as follows: There are three input files, "input", "coordinate", and "in_para".

The file "input" contains the dimension of the simulation space (2 for 2D and 3 for 3D), the average Ti concentration (ϕ), the average V configuration ($5-\phi$), the radius of the initial seeds, the simulation mesh spacing (dx), the simulation time step (dt), the gradient surface energy coefficient (ε), the restart flag (0 for new calculation and 1 for continuing calculation), the time steps at which output file is written (for example, 10000), the maximum time step (for example, 100000), the number of meshes in x direction (Nx), the number of meshes in y direction (Ny), the number of meshes in z direction (Nz), the amplitude of seed (the c parameter), the amplitude for random force, and the coefficient for the polynomial in the free energy boundary at $\phi = 0$. The provided "input" file is for the 3D simulation with $Nx = Ny = Nz = 40$. For example, "input" file for 6.25 at% V for FPPF1 (tetrahedron model) is as follows:

```
3             :: 3D calculation
4.250         :: phi_Ti
0.750         :: phi_V
4.0           :: radius of the seed
0.27d0        :: dx
0.000125d0    :: dt, M = 1.0
0.01          :: the gradient energy coefficient, epsilon_x
0             :: restart
10000         :: time steps interval at which output file is written
100000        :: maximum time step
40            :: Nx
40            :: Ny
40            :: Nz
0.3           :: amplitude of seeds, the c parameter
0.5           :: amplitude for random fluctuation
0.05000       :: coefficient for the polynomial in free energy boundary
```

Numbers (2nd, 3rd) in the second and third lines should be set as $(4.5 - n, n + 0.5)$ for $Ti_{4-n}V_n$. In our simulation, n is chosen as $n = 0.25m$ with $m = 1, 2, ..., 15$. For FPPF2 using a 8-atom unit cell, only the second and third lines are changed as

```
3             :: 3D calculation
8.000         :: phi_Ti
1.000         :: phi_V
...           ...
```

Numbers (2nd, 3rd) in the second and third lines should be set as $(8.5 - n, n + 0.5)$ for $Ti_{8-n}V_n$. In our simulation, n is chosen as $n = 0.5m$ with $m = 1, 2, ..., 15$. Similarly, for FPPF3 using a 16-atom unit cell, the second and third lines become

```
3             :: 3D calculation
15.50         :: phi_Ti
1.500         :: phi_V
...           ...
```

Numbers (2nd, 3rd) in the second and third lines should be set as $(16.5 - n, n + 0.5)$ for $Ti_{16-n}V_n$. In our simulation, n is chosen as $n = 1, 2, ..., 15$.

The file "coordinate" contains the positions of the initial fluctuations together with ±1 for the factor of the c parameter (defined in "input") of the Ti concentration in the direct (fractional) coordinate. (The minus of these initial fluctuations values are set for the V concentration at the same positions.) The number at the top line denotes the number of the $\pm c$ positions to be given below. The positions (the first three columns) should be random numbers between zero and one. "coordinate" file is common for FPPF1, FPPF2, and FPPF3, and is as follows:

```
22
0.93 0.96 0.18 1.00
0.83 0.81 0.71 1.00
0.62 0.11 0.23 1.00
0.08 0.50 0.01 1.00
0.70 0.08 0.63 1.00
0.47 0.04 0.39 1.00
0.76 0.71 0.10 1.00
0.52 0.37 0.49 1.00
0.68 0.81 0.94 1.00
0.01 0.60 0.20 1.00
0.44 0.24 0.90 1.00
0.21 0.88 0.50 -1.00
0.68 0.29 0.45 -1.00
0.46 0.56 0.77 -1.00
0.55 0.77 0.86 -1.00
0.28 0.92 0.41 -1.00
0.38 0.33 0.25 -1.00
0.30 0.11 0.05 -1.00
0.98 0.54 0.76 -1.00
0.85 0.43 0.90 -1.00
0.15 0.12 0.26 -1.00
0.83 0.61 0.44 -1.00
```

The file "in_para" contains the local free energy values for all configurations of Ti_nV_m in a matrix form from $n = m = 0$ to $n = m = natom$, where *natom* is written at the top line of this file. The local free energies that are not explicitly defined by the "in_para" file are defined in the quartic function subroutine "F_En" in Util_mod.f90. In the provided "in_para" file, *natom* is set at 6, which allows at most two interstitial atoms in the unit cell. "in_para" file is different for FPPF1, FPPF2, FPPF3 and for 0K and 1300K. All files are given as follows:

"in_para" file for FPPF1 at 0 K:

```
6
  0.00000000  -3.21428563 -11.34801896 -22.45538470 -34.12510709 -31.52135419 -28.36412594
 -2.95590015 -10.85387055 -21.39255290 -32.33990454 -30.34589100 -26.39185996 -18.00000000
-10.22729416 -20.27765652 -30.51047774 -28.09005080 -24.18942862 -16.00000000  -8.00000000
-18.36938210 -28.66581653 -26.63318377 -22.08551144 -14.00000000  -6.00000000   6.00000000
-27.49000000 -24.23323142 -20.09744355 -12.00000000  -4.00000000   8.00000000  16.00000000
-21.66924278 -17.38847969 -10.00000000  -2.00000000  10.00000000  18.00000000  28.00000000
-14.69787070  -8.00000000   0.00000000  12.00000000  20.00000000  30.00000000  40.00000000
```

"in_para" file for FPPF1 at 1300 K:

```
6
  0.00000000  -3.21428563 -11.34801896 -22.45538470 -34.03632395 -31.52135419 -28.36412594
 -2.95590015 -10.85387055 -21.39255290 -32.27281072 -30.34589100 -26.39185996 -18.00000000
-10.22729416 -20.27765652 -30.45135831 -28.09005080 -24.18942862 -16.00000000  -8.00000000
-18.36938210 -28.59023686 -26.63318377 -22.08551144 -14.00000000  -6.00000000   6.00000000
-27.41000000 -24.23323142 -20.09744355 -12.00000000  -4.00000000   8.00000000  16.00000000
-21.66924278 -17.38847969 -10.00000000  -2.00000000  10.00000000  18.00000000  28.00000000
-14.69787070  -8.00000000   0.00000000  12.00000000  20.00000000  30.00000000  40.00000000
```

“in_para” file for FPPF2 at 0 K:

```
10
  0.0000   -1.6071   -3.2143   -6.4776  -11.3480  -12.8348  -22.4554  -20.3013  -34.1251  -21.4347  -31.5214
 -1.4780   -3.0851   -7.0341  -11.1009  -16.3703  -21.9240  -27.3976  -33.2325  -32.2355  -30.9336  -28.9566
 -2.9559   -6.8128  -10.8539  -15.9680  -21.3926  -26.6746  -32.3399  -31.2252  -30.3459  -28.1121  -26.3919
 -6.5916  -10.5406  -15.5658  -20.8351  -25.9515  -31.4252  -30.2150  -29.2180  -27.2677  -25.2906  -21.1959
-10.2273  -14.9321  -20.2777  -25.2116  -30.5105  -29.3934  -28.0901  -26.1777  -24.1894  -20.1453  -16.0000
-14.2983  -19.3235  -24.4717  -29.5881  -28.5718  -27.3616  -25.0878  -23.1375  -19.0947  -15.0000  -11.0000
-18.3694  -23.7007  -28.6658  -27.5107  -26.6332  -24.2265  -22.0855  -18.0687  -14.0000  -10.0000   -6.0000
-22.9297  -28.0779  -26.4495  -25.4332  -23.3653  -21.0915  -17.0428  -13.0000   -9.0000   -5.0000    1.0000
-27.4900  -25.5146  -24.2332  -22.0881  -20.0974  -16.0457  -12.0000   -8.0000   -4.0000    2.0000    8.0000
-24.5796  -22.9512  -20.8109  -18.7430  -15.0487  -11.0000   -7.0000   -3.0000    3.0000    9.0000   13.0000
-21.6692  -19.4972  -17.3885  -13.8715  -10.0000   -6.0000   -2.0000    4.0000   10.0000   14.0000   18.0000
```

“in_para” file for FPPF2 at 1300 K:

```
10
  0.0000   -1.6071   -3.2143   -6.4776  -11.3480  -12.8348  -22.4554  -20.2570  -34.0363  -21.4347  -31.5214
 -1.4780   -3.0851   -7.0341  -11.1009  -16.3703  -21.9240  -27.3641  -33.1546  -32.1911  -30.9336  -28.9566
 -2.9559   -6.8128  -10.8539  -15.9680  -21.3926  -26.6430  -32.2728  -31.1863  -30.3459  -28.1121  -26.3919
 -6.5916  -10.5406  -15.5658  -20.8351  -25.9220  -31.3621  -30.1814  -29.2180  -27.2677  -25.2906  -21.1959
-10.2273  -14.9321  -20.2777  -25.1780  -30.4514  -29.3619  -28.0901  -26.1777  -24.1894  -20.1453  -16.0000
-14.2983  -19.3235  -24.4339  -29.5208  -28.5423  -27.3616  -25.0878  -23.1375  -19.0947  -15.0000  -11.0000
-18.3694  -23.6618  -28.5902  -27.4770  -26.6332  -24.2265  -22.0855  -18.0687  -14.0000  -10.0000   -6.0000
-22.8897  -28.0001  -26.4117  -25.4332  -23.3653  -21.0915  -17.0428  -13.0000   -9.0000   -5.0000    1.0000
-27.4100  -25.4757  -24.2332  -22.0881  -20.0974  -16.0457  -12.0000   -8.0000   -4.0000    2.0000    8.0000
-24.5396  -22.9512  -20.8109  -18.7430  -15.0487  -11.0000   -7.0000   -3.0000    3.0000    9.0000   13.0000
-21.6692  -19.4972  -17.3885  -13.8715  -10.0000   -6.0000   -2.0000    4.0000   10.0000   14.0000   18.0000
```

“in_para” file for FPPF3 at 0 K:

```
20
  0.0000   -0.8036   -1.6071   -2.4107   -3.2143   -3.4397   -6.0758   -8.7119  -11.3480   -6.8192  -12.0313  -17.2433  -22.4554  -10.3393  -18.2679  -26.1965  -34.1251  -10.2911  -17.3678  -24.4446  -31.5214
 -0.7390   -1.5425   -2.3461   -3.1497   -5.1242   -5.6122   -8.4184  -11.2245  -13.8592  -11.0948  -16.6423  -22.1897  -24.9265  -18.4142  -26.0465  -33.6788  -33.1803  -18.1758  -24.7017  -31.2275  -30.2390
 -1.4780   -2.2815   -3.0851   -5.0366   -7.0341   -8.3257  -11.1009  -13.6968  -16.3703  -17.2142  -21.9240  -24.6129  -27.3976  -26.1835  -33.2325  -32.7046  -32.2355  -24.9587  -30.9336  -29.8809  -28.9566
 -2.2169   -3.0205   -4.9490   -6.9235   -8.9440  -10.9774  -13.5345  -16.1692  -18.8814  -21.6583  -24.2993  -27.0361  -29.8688  -32.7862  -32.2289  -31.7304  -31.2907  -30.6398  -29.5229  -28.5344  -27.6742
 -2.9559   -4.8614   -6.8128   -8.8103  -10.8539  -13.3722  -15.9680  -18.6415  -21.3926  -23.9857  -26.6746  -29.4593  -32.3399  -31.7532  -31.2252  -30.7561  -30.3459  -29.1648  -28.1121  -27.1878  -26.3919
 -4.7737   -6.7022   -8.6767  -10.6972  -13.2098  -15.7669  -18.4016  -21.1138  -23.6720  -26.3130  -29.0499  -31.8825  -31.2774  -30.7201  -30.2216  -29.7819  -28.8068  -27.6899  -26.7014  -25.8413  -23.7939
 -6.5916   -8.5431  -10.5406  -12.9731  -15.5658  -18.1616  -20.8351  -23.3477  -25.9515  -28.6404  -31.4252  -30.8434  -30.2150  -29.6871  -29.2180  -28.2523  -27.2677  -26.2150  -25.2906  -23.2559  -21.1959
 -8.4094  -10.3839  -12.7363  -15.2489  -17.9217  -20.5564  -23.0234  -25.5816  -28.2310  -30.9678  -30.4093  -29.8042  -29.1525  -28.6540  -27.6978  -26.7227  -25.7285  -24.7400  -22.7180  -20.6706  -18.5980
-10.2273  -12.4996  -14.9321  -17.5247  -20.2777  -22.6990  -25.2116  -27.8154  -30.5105  -29.9752  -29.3934  -28.7650  -28.0901  -27.1434  -26.1777  -25.1931  -24.1894  -22.1800  -20.1453  -18.0853  -16.0000
-12.2628  -14.6152  -17.1278  -19.8006  -22.3747  -24.8417  -27.3999  -30.0493  -29.5412  -28.9826  -28.3775  -27.7258  -26.5889  -25.6328  -24.6576  -23.6634  -21.6421  -19.6200  -17.5727  -15.5000  -13.5000
-14.2983  -16.7308  -19.3235  -21.9434  -24.4717  -26.9843  -29.5881  -29.0453  -28.5718  -27.9900  -27.3616  -26.1915  -25.0878  -24.1221  -23.1375  -21.1226  -19.0947  -17.0600  -15.0000  -13.0000  -11.0000
-16.3339  -18.8465  -21.5121  -24.0862  -26.5688  -29.1270  -28.5494  -28.0413  -27.6025  -26.9974  -25.7941  -24.6572  -23.5866  -22.6115  -20.6031  -18.5817  -16.5474  -14.5000  -12.5000  -10.5000   -8.5000
-18.3694  -21.0808  -23.7007  -26.2290  -28.6658  -28.0535  -27.5107  -27.0372  -26.6332  -25.3967  -24.2265  -23.1228  -22.0855  -20.0836  -18.0687  -16.0409  -14.0000  -12.0000  -10.0000   -8.0000   -6.0000
-20.6495  -23.3152  -25.8893  -28.3719  -27.5577  -26.9801  -26.4719  -26.0332  -24.9992  -23.7959  -22.6590  -21.5885  -19.5641  -17.5557  -15.5344  -13.5000  -11.5000   -9.5000   -7.5000   -5.5000   -2.5000
-22.9297  -25.5496  -28.0779  -27.1770  -26.4495  -25.9067  -25.4332  -24.3799  -23.3653  -22.1952  -21.0915  -19.0664  -17.0428  -15.0279  -13.0000  -11.0000   -9.0000   -7.0000   -5.0000   -2.0000    1.0000
-25.2098  -27.7840  -26.7962  -25.9820  -25.3414  -24.8332  -23.7606  -22.7267  -21.7314  -20.5945  -18.5686  -16.5442  -14.5214  -12.5000  -10.5000   -8.5000   -6.5000   -4.5000   -1.5000    1.5000    4.5000
-27.4900  -26.4155  -25.5146  -24.7871  -24.2332  -23.1413  -22.0881  -21.0735  -20.0974  -18.0708  -16.0457  -14.0221  -12.0000  -10.0000   -8.0000   -6.0000   -4.0000   -1.0000    2.0000    5.0000    8.0000
-26.0348  -25.0471  -24.2329  -23.5922  -22.5220  -21.4495  -20.4155  -19.4202  -17.5731  -15.5472  -13.5229  -11.5000   -9.5000   -7.5000   -5.5000   -3.5000   -0.5000    2.5000    5.5000    8.5000   10.5000
-24.5796  -23.6787  -22.9512  -21.8731  -20.8109  -19.7576  -18.7430  -16.9402  -15.0487  -13.0236  -11.0000   -9.0000   -7.0000   -5.0000   -3.0000  -13.3581    3.0000    6.0000    9.0000   11.0000   13.0000
-23.1244  -22.3102  -21.2242  -20.1540  -19.0997  -18.0657  -16.3072  -14.4601  -12.5244  -10.5000   -8.5000   -6.5000   -4.5000   -2.5000    0.5000    3.5000    6.5000    9.5000   11.5000   13.5000   15.5000
-21.6692  -20.5753  -19.4972  -18.4349  -17.3885  -15.6743  -13.8715  -11.9801  -10.0000   -8.0000   -6.0000   -4.0000   -2.0000    1.0000    4.0000    7.0000   10.0000   12.0000   14.0000   16.0000   18.0000
```

“in_para” file for FPPF3 at 1300 K:

```
20
  0.0000   -0.8036   -1.6071   -2.4107   -3.2143   -3.4397   -6.0758   -8.7119  -11.3480   -6.8192  -12.0313  -17.2433  -22.4554  -10.3171  -18.2235  -26.1299  -34.0363  -10.2911  -17.3678  -24.4446  -31.5214
 -0.7390   -1.5425   -2.3461   -3.1497   -5.1242   -5.6122   -8.4184  -11.2245  -13.8592  -11.0948  -16.6423  -22.1897  -24.9097  -18.3726  -25.9840  -33.5954  -33.1137  -18.1758  -24.7017  -31.2275  -30.2390
 -1.4780   -2.2815   -3.0851   -5.0366   -7.0341   -8.3257  -11.1009  -13.6968  -16.3703  -17.2142  -21.9240  -24.5966  -27.3641  -26.1251  -33.1546  -32.6421  -32.1911  -24.9587  -30.9336  -29.8809  -28.9566
 -2.2169   -3.0205   -4.9490   -6.9235   -8.9440  -10.9774  -13.5345  -16.1692  -18.8814  -21.2835  -24.2993  -27.0036  -29.8185  -32.7137  -32.1704  -31.6887  -31.2685  -30.6398  -29.5229  -28.5344  -27.6742
 -2.9559   -4.8614   -6.8128   -8.8103  -10.8539  -13.3722  -15.9680  -18.6415  -21.3926  -23.9704  -26.6430  -29.4105  -32.2728  -31.6988  -31.1863  -30.7353  -30.3459  -29.1648  -28.1121  -27.1878  -26.3919
 -4.7737   -6.7022   -8.6767  -10.6972  -13.2098  -15.7669  -18.4016  -21.1138  -23.6573  -26.2825  -29.0026  -31.8174  -31.2271  -30.6838  -30.2021  -29.7819  -28.8068  -27.6899  -26.7014  -25.8413  -23.7939
 -6.5916   -8.5431  -10.5406  -12.9731  -15.5658  -18.1616  -20.8351  -23.3319  -25.9220  -28.5946  -31.3621  -30.7945  -30.1814  -29.6689  -29.2180  -28.2523  -27.2677  -26.2150  -25.2906  -23.2559  -21.1959
 -8.4094  -10.3839  -12.7363  -15.2489  -17.9217  -20.5564  -23.0065  -25.5500  -28.1867  -30.9067  -30.3620  -29.7716  -29.1357  -28.6540  -27.6978  -26.7227  -25.7285  -24.7400  -22.7180  -20.6706  -18.5980
-10.2273  -12.4996  -14.9321  -17.5247  -20.2777  -22.6812  -25.1780  -27.7680  -30.4514  -29.9294  -29.3619  -28.7487  -28.0901  -27.1434  -26.1777  -25.1931  -24.1894  -22.1800  -20.1453  -18.0853  -16.0000
-12.2628  -14.6152  -17.1278  -19.8006  -22.3550  -24.8059  -27.3494  -29.9861  -29.4968  -28.9521  -28.3617  -27.7258  -26.5889  -25.6328  -24.6576  -23.6634  -21.6421  -19.6200  -17.5727  -15.5000  -13.5000
-14.2983  -16.7308  -19.3235  -21.9242  -24.4339  -26.9307  -29.5208  -28.9979  -28.5423  -27.9747  -27.3616  -26.1915  -25.0878  -24.1221  -23.1375  -21.1226  -19.0947  -17.0600  -15.0000  -13.0000  -11.0000
-16.3339  -18.8465  -21.4927  -24.0479  -26.5121  -29.0555  -28.4989  -28.0096  -27.5877  -26.9974  -25.7941  -24.6572  -23.5866  -22.6115  -20.6031  -18.5817  -16.5474  -14.5000  -12.5000  -10.5000   -8.5000
-18.3694  -21.0611  -23.6618  -26.1715  -28.5902  -27.9999  -27.4770  -27.0214  -26.6332  -25.3967  -24.2265  -23.1228  -22.0855  -20.0836  -18.0687  -16.0409  -14.0000  -12.0000  -10.0000   -8.0000   -6.0000
-20.6295  -23.2758  -25.8310  -28.2952  -27.5010  -26.9444  -26.4551  -26.0332  -24.9992  -23.7959  -22.6590  -21.5885  -19.5641  -17.5557  -15.5344  -13.5000  -11.5000   -9.5000   -7.5000   -5.5000   -2.5000
-22.8897  -25.4904  -28.0001  -27.1194  -26.4117  -25.8888  -25.4332  -24.3799  -23.3653  -22.1952  -21.0915  -19.0664  -17.0428  -15.0279  -13.0000  -11.0000   -9.0000   -7.0000   -5.0000   -2.0000    1.0000
-25.1498  -27.7051  -26.7379  -25.9437  -25.3225  -24.8332  -23.7606  -22.7267  -21.7314  -20.5945  -18.5686  -16.5442  -14.5214  -12.5000  -10.5000   -8.5000   -6.5000   -4.5000   -1.5000    1.5000    4.5000
-27.4100  -26.3564  -25.4757  -24.7680  -24.2332  -23.1413  -22.0881  -21.0735  -20.0974  -18.0708  -16.0457  -14.0221  -12.0000  -10.0000   -8.0000   -6.0000   -4.0000   -1.0000    2.0000    5.0000    8.0000
-25.9748  -25.0076  -24.2135  -23.5922  -22.5220  -21.4495  -20.4155  -19.4202  -17.5731  -15.5472  -13.5229  -11.5000   -9.5000   -7.5000   -5.5000   -3.5000   -0.5000    2.5000    5.5000    8.5000   10.5000
-24.5396  -23.6589  -22.9512  -21.8731  -20.8109  -19.7576  -18.7430  -16.9402  -15.0487  -13.0236  -11.0000   -9.0000   -7.0000   -5.0000   -3.0000  -13.3581    3.0000    6.0000    9.0000   11.0000   13.0000
-23.1044  -22.3102  -21.2242  -20.1540  -19.0997  -18.0657  -16.3072  -14.4601  -12.5244  -10.5000   -8.5000   -6.5000   -4.5000   -2.5000    0.5000    3.5000    6.5000    9.5000   11.5000   13.5000   15.5000
-21.6692  -20.5753  -19.4972  -18.4349  -17.3885  -15.6743  -13.8715  -11.9801  -10.0000   -8.0000   -6.0000   -4.0000   -2.0000    1.0000    4.0000    7.0000   10.0000   12.0000   14.0000   16.0000   18.0000
```

On the other hand, the simulation code for the continuous phase-field (CPF) model can also be downloaded from the website [2]. The values of A, B, C, and D at 0 K are set in the function subprogram, F_En(xx,c1), of Util_cpf.f90 as

A = -26.7390818d0
B = -34.16063283d0
C = -27.49d0
D = 0.05d0

The values of A, B, and C should be changed by adding the renormalization correction ΔF for the simulation at 1300 K as

A = -26.7390818d0 + 0.08229089d0
B = -34.16063283d0 + 0.08878314d0
C = -27.49d0 + 0.078d0
D = 0.05d0

The code can be compiled by using Makefile_cpf. The usage of this code is as follows: There are three input files, "input", "coordinate", and "in_para", although any content of "in_para" is not used in the actual calculation in the CPF method. The files "input" and "coordinate" is the same as that used for FPPF1.

References

[1] T. N. Pham, K. Ohno, R. Sahara, R. Kuwahara, S. Bhattacharyya, *Clear evidence of element partitioning effects in a Ti-6Al-4V alloy by the first-principles phase field method*, J. Phys.: Cond. Mat. 32, 264001(pp.9) (2020). doi: 10.1088/1361-648X/ab7ad5

[2] https://pf.tombo.page/

[3] S. Bhattacharyya, R. Sahara, and K. Ohno, *A first-principles phase field method for quantitatively predicting multi-composition phase separation without thermodynamic empirical parameter*, Nature Communications 10, 3451 (pp.10) (2019). doi:10.1038/s41467-019-11248-z
